# *Euphorbia humifusa* Willd exerts inhibition of breast cancer cell invasion and metastasis through inhibition of TNFα-induced MMP-9 expression

**DOI:** 10.1186/s12906-016-1404-6

**Published:** 2016-10-24

**Authors:** Soon Young Shin, Chang Gun Kim, You Jung Jung, Yearam Jung, Hyeryoung Jung, Jihyun Im, Yoongho Lim, Young Han Lee

**Affiliations:** 1 Department of Biological Sciences, College of Biological Science and Biotechnology, Konkuk University, Seoul, Republic of Korea; 2 Cancer and Metabolism Institute, Konkuk University, Seoul, Republic of Korea; 3 Division of Bioscience and Biotechnology, College of Biological Science and Biotechnology, BMIC, Konkuk University, Seoul, Republic of Korea; 4 College of Biological Science and Biotechnology, Konkuk University, 120 Neundong-ro, Gwangjin-gu, Seoul, 05029 Republic of Korea

**Keywords:** *Euphorbia humifusa* Willd, Invasion, Metastasis, Matrix metalloproteinase-9, Tumor necrosis factor alpha, Nuclear factor-kappa B

## Abstract

**Background:**

Breast cancer is the most common type of malignancy in women worldwide. *Euphorbia humifusa* Willd (EuH) is a plant that is widely used as a traditional medicine. However, no systemic studies on the anti-cancer effects of EuH have been reported. The aim of this study is to evaluate the anti-metastatic effect of the EuH.

**Methods:**

Ethyl acetate fraction was prepared from EuH methanol extracts (EA/EuH). Inhibitory effect of EA/EuH on cell migration was determined using an in vitro scratch-wound healing assay. The anti-invasive activity was determined by in vitro three-dimensional spheroid culture system and in vivo syngenic experimental lung metastasis experiment. Gene expression profiles were analyzed by using RT-PCR, real-time PCR, and luciferase reporter assay systems.

**Results:**

Ethyl acetate fraction from the EuH extract (EA/EuH) inhibited the migration and invasive capabilities of highly metastatic MDA-MB-231 breast cancer cells and attenuated syngeneic lung metastasis of mouse 4 T1 breast cancer cells in vivo. Mechanistically, EA/EuH decreased tumor necrosis factor alpha (TNFα)-induced matrix metalloproteinase (MMP)-9 mRNA expression through the inhibition of NF-κB activity in MDA-MB-231 cells.

**Conclusion:**

EuH may be beneficial in the prevention of invasion and metastasis of early stage breast cancer and can be served as an anti-metastatic agent or adjuvant therapy against metastatic breast cancer.

## Background

Breast cancer is the most common type of malignancy in women worldwide, accounting for approximately 23 % of total cancer cases and 14 % of total cancer-related deaths [1]. Breast cancer cells often exhibit high invasive and metastatic properties. Metastasis is the uncontrolled spread of primary tumor cells to other sites in the body and commonly occurs in the late stages of cancer. Like other cancer types, the prognosis of breast cancer patients is strongly influenced by the stage of metastasis.

The tumor microenvironment is the cellular environment that surrounds the tumor site, and is composed of extracellular matrix (ECM), blood vessels, and various cell types, including stromal fibroblasts and infiltrative immune cells. Tumor necrosis factor alpha (TNFα) is a pro-inflammatory cytokine involved in the modulation of systemic inflammation. In the tumor microenvironment, TNFα is produced by tumor cells as well as tumor-associated cells and plays a central role in promoting tumor invasion and metastasis [2]. During malignant progression, breast epithelial cells undergo a transition to mesenchymal-type cells (epithelial-to-mesenchymal transition). These cells are migratory and can invade through the surrounding ECM. Matrix metalloproteinases (MMPs) are zinc-dependent proteases that are mainly involved in tissue remodelling via various physiological and pathological processes that degrade ECM proteins. Emerging evidence has emphasized the role of MMPs in migration and invasion of cancer cells through the breakdown of ECM and basement membranes [3].

The transcription factor NF-κB regulates many genes in control of inflammatory responses, cell proliferation, cell survival, angiogenesis, and metastasis [4]. The NF-κB family consists of five members, including NF-κB1 (also called p50), NF-κB2 (also called p52), RelA (also called p65), RelB, and c-Rel [5]. In unstimulated cells, NF-κB is sequestered in the cytoplasm by the inhibitory protein IκB, which hinders the nuclear localization of NF-κB. Upon stimulation of the cells, IκB kinases (IKK) stimulate NF-κB through the phosphorylation of IκB. This results in a degradation and dissociation of IκB from NF-κB leading to the translocation of NF-κB into the nucleus. In many different types of human cancers, a constitutively activated NF-κB is common [6]. Aberrant activation of NF-κB is known to be associated with the progression of breast cancer [7], particularly the promotion of tumor cell invasion, migration, and metastasis through the upregulation of MMP-9 expression [8]. Therefore, it seems likely that the inhibition of NF-κB activity is required for the prevention and treatment of cancer. It should be noted that several agents inhibiting NF-κB functions are currently in clinical use or undergoing clinical development as cancer chemotherapeutics [4, 9–11].

*Euphorbia humifusa* Willd (EuH), known as Ttang-Bin-Dae in Korea or Di-Jin-Cao in China, is a dicotyledonous, polypetalous plant widely distributed in eastern Asia. In Korea, EuH has been used as a traditional medicinal plant for the treatment of diarrhoea, jaundice, dysentery, enteritis, diabetes, and asthma [12, 13]. Previous phytochemical studies have isolated multiple bioactive compounds from the EuH, including euphorbinoside dehydropicrorhiza acid methyl diester, flavone glucosides, apigenin glycosides, tannins, α-pyrrolidinonoids, lanostane triterpenoids [14–16], exhibiting various pharmacological actions, including antifungal, anti-inflammatory, vasorelaxant, and anti-viral properties [12, 16–18].

However, the effects of EuH on cancer progression, such as invasion and metastasis, have not been elucidated. In this study, we examined the anti-metastatic effects of EuH on the highly metastatic human breast cancer cell line MDA-MB-231. Our results indicate that EuH inhibits migration, invasion, and experimental metastasis of MDA-MB-231 cells. Furthermore, EuH reduces TNFα-induced MMP-9 expression through the inhibition of NF-κB activity. The data suggest that EuH may be beneficial in the prevention of an invasion and metastasis of early stage breast cancer.

## Methods

### Cells and chemicals

Human breast carcinoma cells (MDA-MB-231, MDA-MB-435, T47D, and MCF7) were obtained from the American Type Culture Collection (ATCC, Manassas, VA, MD, USA). Mouse mammary carcinoma cells (4 T1) were kindly provided by Dr. Jeong-Seok Nam (Gwangju Institute of Science and Technology, Gwangju, Korea). Cells were grown in Dulbecco’s modified Eagle’s medium (Corning Cellgro, Manassas, VA, USA) supplemented with 10 % (v/v) heat-inactivated foetal bovine serum (Corning Cellgro). TNFα, methanol, n-hexane, chloroform, ethyl acetate, n-butanol, dimethylsulfoxide (DMSO), Tris-base, HEPES, Triton X-100, glycerol, leupeptin, phenylmethylsulfonyl fluoride (PMSF) were purchased from Sigma-Aldrich (Saint Louis, MO, USA). SDS, acrylamide, bisacrylamide, ammoniuk persulfate, nitrocellular membranes were from Bio-Rad Laboratories (Hercules, CA, USA). Pierce™ BCA Protein Assay Reagent was obtained from Thermo Scientific (Rockford, IL, USA).

### Preparation of the *E. humifusa* Willd extracts

Air dried *E. humifusa* Willd (EuH) was purchased from an herbal market from Gyungdong Pharmaceutical Market (Seoul, Republic of Korea) and taxonomically identified by a galenical pharmacist, Dr. Hi Jae Cho (College of Biological Science and Biotechnology, Konkuk University, Seoul, Republic of Korea). A voucher specimen was deposited at the College of Biological Science and Technology, Konkuk University, Korea. EuH (3 kg) was soaked in methanol for 3 days. The methanolic extract (130 g) was obtained under reduced pressure using a rotary evaporator. By polarity based fractionation, five fractions were collected from 35.93 g of methanol extract: n-hexane (10.08 g; 28.1 %), chloroform (1.36 g; 3.8 %), ethyl acetate (4.91 g; 13.7 %), n-butanol (8.48 g; 23.6 %), and aqueous fraction (11.12 g; 30.9 %). Each fraction was dried using a freeze-dryer and dissolved in DMSO at 10 mg/mL.

### Cell migration assay

Migration of MDA-MB-231 cells was determined using an in vitro scratch-wound healing assay, as described previously [19]. Migrated cells were photographed with an EVOS® FL Auto Cell Imaging System (Life Technologies, Carlsbad, CA, USA). Cells migrated into the gap area in (A) were quantified in a field of view using ImageJ software (https://imagej.nih.gov/ij/; Center for Information Technology, National Institute of Health, Bethesda, MA, USA).

### Actin reorganization

Polymerized F-actin was examined using the Rhodamine-phalloidin-based F-Actin Visualization Biochem Kit (Cytoskeleton, Inc.; Denver, CO, USA), according to the manufacturer’s instructions. Images were captured using an EVOS FL Auto Cell Imaging System (Life Technologies).

### Three-dimensional spheroid culture and invasion assay

Three-dimensional invasion assay was performed using a Cultrex 3-D Spheroid Cell Invasion Assay kit (Trevigen, Inc., Gaithersburg, MD, USA), as described previously [20]. Invasive protrusions into ECM were visualized an EVOS FL Auto Cell Imaging System.

### Syngenic experimental lung metastasis assay

Six-week-old female Balb/c mice were purchased from YoungBio (Seongnam, Gyeonggi-do, Korea). All animal experiments were conducted following the standards and procedures approved by the Konkuk University Institutional Animal Care and Use Committee (No.KU15194). 4 T1 mammary carcinoma cells (6 × 10^4^ cells/50 μL) were injected into the spleen as described previously [21]. One-day after intra-splenic implantation of tumor cells, PBS (*n* = 5) or EA/EuH (10 mg/kg; *n* = 7) was administered daily intraperitoneally. Mice were sacrificed after 8 days of cell inoculation, and lung tissues were stained by routine hematoxylin and eosin (H&E).

### RT-PCR and quantitative real-time PCR

Total RNA was extracted using Isol-RNA lysis reagent (NucleoZOL; Clontech, Mountain View, CA, USA), and the synthesis of cDNA was carried out using an iScript cDNA synthesis kit (Bio-Rad, Hercules, CA, USA). RT-PCR was performed as described previously [22]. For quantitative real-time PCR, TaqMan-iQ supermix kit (Bio-Rad) was used with the Bio-Rad iCycler iQ thermal cycler according to the manufacturer’s instruction. The TaqMan fluorogenic probes and gene-specific PCR primers for MMP-9 and glyceraldehyde-3-phosphate dehydrogenase (GAPDH) were described previously [22]. The relative fold changes were normalized to GAPDH mRNA in the same sample.

### NF-κB-dependent transcriptional activity assay

MDA-MB-231 cells were transfected with 0.1 μg of the 5 × NFκB-Luc plasmid, containing five repeats of NF-κB binding sites and treated with 10 ng/mL TNFα in the absence and presence of EA/EuH, as described previously [20]. The luciferase activities were measured with a Centro LB960 luminometer (Berthold Technologies; Bad Wildbad, Germany).

### Immunoblot analysis

Cells were lysed in 20 mM HEPES (pH 7.2), 1 % (v/v) Triton X-100, 10 % (v/v) glycerol, 150 mM NaCl, 10 μg/mL leupeptin, and 1 mM PMSF. Protein extracts (20 μg per sample) were separated via 10 % SDS-PAGE, transferred to nitrocellulose membranes, and incubated with appropriate primary and secondary antibodies. Primary antibodies against phospho-IκB (Ser32) and phospho-RelA/p65 (Ser536) were obtained from Cell Signaling Technology (Beverly, MA, USA), and an antibody against GAPDH was from Santa Cruz Biotechnology (Dallas, TX, USA). The blots were developed using an enhanced chemiluminescence detection system (GE Healthcare, Piscataway, NJ, USA). The relative protein band intensities were determined using ImageJ software.

### Immunofluorescence microscopy

MDA-MB-231 cells plated on coverslips were treated with or without 10 ng/mL TNFα in the absence and presence of EA/EuH (5 μg/mL) for 30 min, followed by fixation, permeabilization, and incubation of primary antibodies specific to α-tubulin and phospho-p65/RelA (Ser536) for 2 h, as described previously [23]. Fluorescent staining cells were examined under an EVOS FL fluorescence microscope (Advanced Microscopy Group; Bothell, WA, USA).

### MMP-9 promoter reporter assay

Construction of the MMP-9 promoter reporters, wild-type pMMP9-Luc(−925/+13) and disrupted NF-κB binding site pMMP9-Luc(−925/+13)mtNFκB, was described previously [24]. Transfection of promoter reporters and luciferase reporter assay were described previously [24]. Luminescence was measured using a Centro LB960 dual luminometer (Berthold Technologies).

### Statistical analysis

All experiments were performed in triplicate. Data are presented as the mean ± SD. Statistical analysis was performed by one-way ANOVA followed by Sidak’s multiple comparisons test using GraphPad Prism version 7.0 software (GraphPad Software Inc., La Jolla, CA). *P*-values < 0.05 were considered statistically significant.

## Results and discussion

### Ethyl acetate fraction of EuH (EA/EuH) inhibits TNFα-induced motility of MDA-MB-231 breast cancer cells

**Fig. 1:**
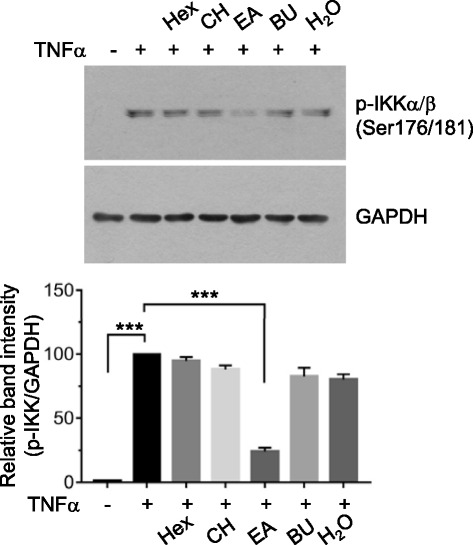
Effect of *E. humifusa* Willd (EA/EuH) extracts on TNFα-induced IKK phosphorylation. Serum-starved MDA-MB-231 cells were left untreated or treated with n-hexane (Hex), chloroform (CH), ethylacetate (EA), n-butanol (Bu), or aqueous (H_2_O) fraction (each 5 μg/mL) for 30 min before stimulation with TNFα (10 ng/mL). After 60 min, whole-cell lysates were prepared, and immunoblotting was performed using the phospho-specific antibody against IKKα/β (Ser176/181). The anti-GAPDH antibody was used as an internal control. Band intensities were analyzed using the ImageJ software. TNFα alone treated-band intensities were set to 100 % (control). The relative values of % of control were plotted as the mean ± SD. *P* values were analyzed by Sidak’s test. (*n* = 3)

NF-κB is a transcription factor found in almost all animal cells and plays a key role in regulating cellular responses, including immune responses, cell proliferation, and survival in diverse cell types [25]. Aberrant activation of NF-κB has been implicated in the progression of breast cancer [7]. Among the NF-κB family, RelA/p65 and p50 complex is the most common form studied intensively in human cells. Upon stimulation, IκB kinase (IKK) activates NF-κB via the phosphorylation of the IκB on serine-32, leading to the proteolysis of IκB. TNFα has been shown to promote cellular migration in the tumor microenvironment [26]. As TNFα stimulates the IKK in various cells, we tested the effect of each EuH extract, including n-hexane, chloroform, ethyl acetate, n-butanol, and aqueous fraction (each 5 μg/mL), on TNFα-induced IKK phosphorylation in highly metastatic MDA-MB-231 human breast cancer cells. Immunoblot analysis shows that the ethyl acetate fraction of EuH (EA/EuH) significantly inhibited TNFα-induced IKK phosphorylation (*P* < 0.0001 by Sidak’s test) (Fig. 1). We choose the EA/EuH for further investigation of the anti-metastatic activity.

**Fig. 2:**
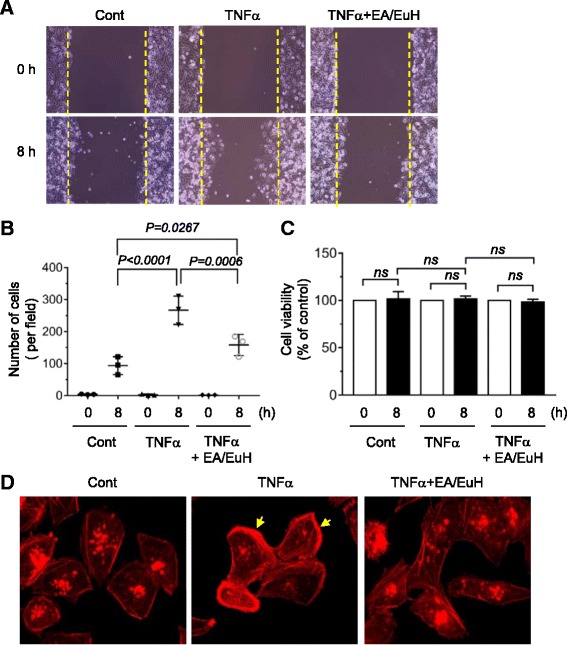
Effect of ethyl acetate fraction of *E. humifusa* Willd (EA/EuH) on TNFα-induced motility of MDA-MB-231 cells. **a** Cell migration assay. MDA-MB-231 cells were treated with or without EA/EuH (5 μg/mL) for 30 min, followed by exposure to TNFα (10 ng/mL). After 8 h, the representative field images were captured by an EVOS FL Auto Cell Imaging System. Dotted lines indicate the scraped boundaries at the beginning of the experiment. **b** Cells migrated into the gap area were quantified in a field of view using ImageJ software. The data shown represent the mean ± SD (*n* = 3). *P* value was analyzed by Sidak’s test. **c** Cell viability assay. MDA-MB-231 cells were treated with or without EA/EuH (5 μg/mL) for 30 min, followed by addition of TNFα (10 ng/mL). After 8 h, viable cells were determined using a Cell Counting Kit-8 (CCK-8). The data shown represent the mean ± SD. *P* value was analyzed by Sidak’s test. *ns*, not-significant. **d** MDA-MB-231 cells were treated with or without EA/EuH (5 μg/mL) for 30 min, followed by exposure to TNFα (10 ng/mL). After 12 h, the cells were stained with rhodamine-phalloidin (1:100) for 1 h and actin rearrangement was analyzed. Arrows indicate polarized F-actin

Cell motility is important for tumor cell invasion and metastasis. To determine whether EA/EuH inhibits TNFα-induced migration of highly metastatic MDA-MB-231 cells, in vitro scratch wound-healing assay was carried out. After scratching confluent monolayers of MDA-MB-231 cells to create a wound-like gap, the cells were treated with TNFα in the absence or presence of 5 μg/mL EA/EuH. TNFα-treated cells efficiently migrated into the gap area compared to untreated cells (Fig. 2a). However, in the presence of EA/EuH, TNFα-induced migration of MDA-MB-231 cells was significantly inhibited (Fig. 2b; *P* = 0.0006 by Sidak’s test). When MDA-MB-231 cells were treated with 5 μg/mL EA/EuH for 8 h, cell viability was not affected by treatment with EA/EuH (Fig. 2c), suggesting that EA/EuH effect on the inhibition of cell migration was not due to its cytotoxicity.

Actin cytoskeletal reorganization plays a key role in the regulation of cellular movement [27]. During cell migration, globular actin monomer (G-actin) assembles to form helical filamentous actin (F-actin) bundles and networks. To determine whether EA/EuH affects actin reorganization, F-actin bundle formation was examined by rhodamine-labelled phalloidin, a class of phallotoxins that binds selectively to F-actin. MDA-MB-231 cells treated with 10 ng/mL TNFα displayed a polarized pattern of F-actin distributed mostly at cell edge, which was substantially reduced by 5 μg/mL EA/EuH (Fig. 2d). These data demonstrate that EA/EuH attenuates TNFα-induced migratory activity of MDA-MB-231 cells.

### EA/EuH inhibits TNFα-induced invasive capability of MDA-MB-231 breast cancer cells

**Fig. 3:**
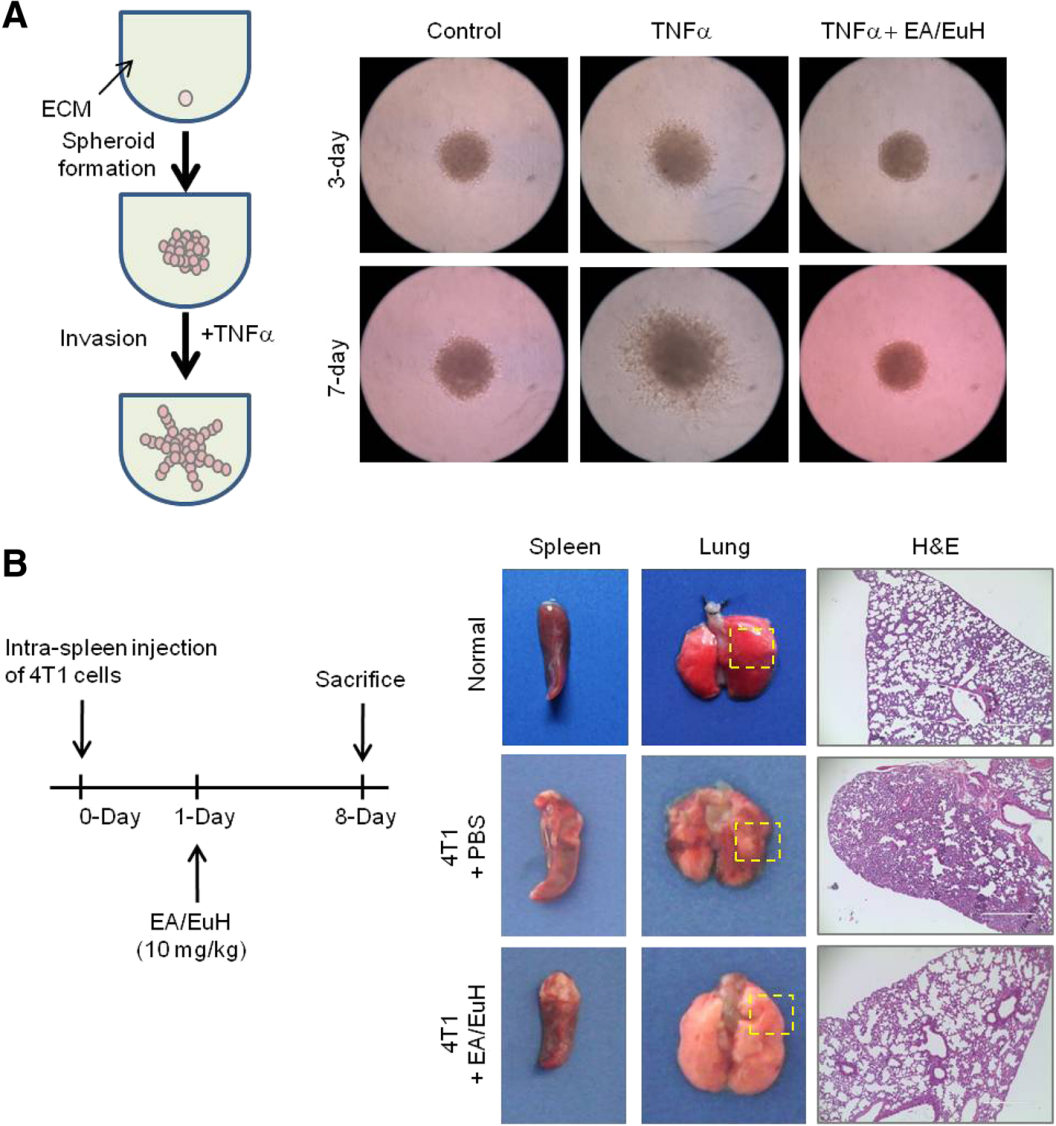
Inhibitory effect of EA/EuH on the invasive capability of MDA-MB-231 cells and experimental lung metastasis of 4 T1 cells. **a** MDA-MB-231 cell spheroids in the extracellular matrix were either untreated (control) or treated with TNFα (10 ng/mL) in the absence or presence of EA/EuH (5 μg/mL). Morphology of 3-D cell invasion was captured at 3-days and 7-days with an EVOS FL Auto Cell Imaging System. **b** Mouse 4 T1 mammary tumor cells were implanted into the spleen of Balb/c mouse. At 1 day post intra-splenic implantation of 4 T1 cells, either phosphate buffered saline (PBS) or EA/EuH (10 mg/kg) was administered intraperitoneally daily. After 8 days, mice were sacrificed. Lung tissues were fixed and stained with hematoxylin and eosin (H&E)

In addition to cell migration, invasiveness into the surrounding ECM is a critical feature of metastatic cancer cells. To evaluate the inhibitory effect of EA/EuH on tumor invasion, 3-D spheroids of MDA-MB-231 cells were formed in an ECM-like environment and then monitored for their invasive capability. As shown in Fig. 3a, the MDA-MB-231 spheroids remained non-invasive under unstimulated conditions. After 3 days of 10 ng/mL TNFα stimulation, the cells began to spread out of the spheroid into the surrounding matrix with a typical starburst pattern. However, in the presence of 5 μg/mL EA/EuH, TNFα-induced invasive protrusion was almost absent. These data suggest that EA/EuH could inhibit the invasive capability of breast cancer cells.

### EA/EuH reduces experimental lung metastasis of 4 T1 mouse mammary tumor cells in vivo

We next investigated whether EA/EuH inhibits tumor metastasis in vivo. Since tumor metastatic progression is strongly influenced by host immune responses and the interaction of tumor cells with tumor microenvironment, we employed a mouse syngeneic tumor metastasis model. 4 T1 mouse mammary carcinoma cells are highly invasive and can metastasize to multiple sites, including the lung [28]. We implanted 4 T1 cells into the spleen of Balb/c mouse (*n* = 15), and they were allowed to form tumors. After 1 day, we randomized the mice into two groups, with one control group receiving phosphate buffered saline (PBS) (*n* = 5) and the other receiving 10 mg/kg EA/EuH (*n* = 7) daily by intraperitoneal injection. Eight days after implantation, all mice were sacrificed. As shown in Fig. 3b, mice that received EA/EuH showed smaller metastatic foci in the lungs as compared to control group mice. These data suggest that EA/EuH has a potent inhibitory effect against experimental lung metastasis.

### EA/EuH suppresses TNFα-induced MMP-9 mRNA expression

For migration and invasion of tumor cells, proteolytic disruption of basement membranes and ECM proteins is critical [3]. MMP-9 degrades collagen in the basement membrane and ECM. MMP-9 levels are closely linked to the promotion of tumor invasion and metastasis, and specific inhibitors of MMP have been shown to inhibit tumor cell invasion [29]. Indeed, MMP-9 is overexpressed in breast cancers and is associated with the promotion of metastasis [30].

**Fig. 4:**
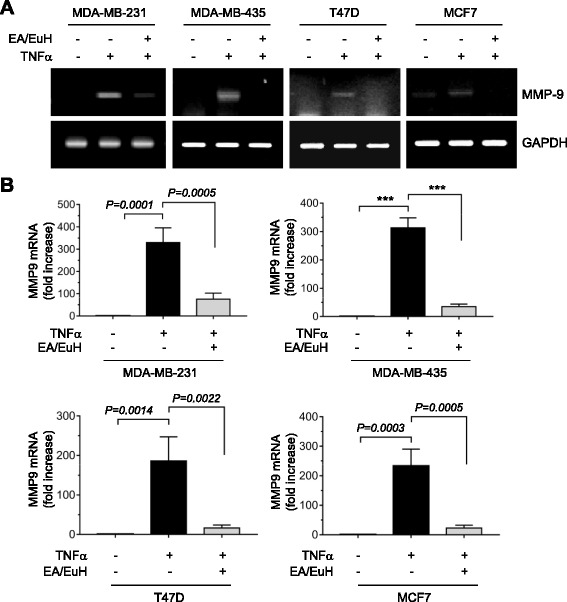
Inhibitory effect of EA/EuH on TNFα-induced MMP-9 mRNA expression. **a** RT-PCR. MDA-MB-231, MDA-MB-435, T47D, and MCF7 cells were treated with or without TNFα (10 ng/mL) in the absence of presence of EA/EuH (5 μg/mL) for 18 h. Total RNA was isolated and then RT-PCR was performed. GAPDH mRNA was used as an internal control. **b** Real-time PCR. MDA-MB-231, MDA-MB-435, T47D, and MCF7 cells were treated as in (**a**). MMP-9 mRNA levels were measured by quantitative real-time PCR. The relative fold changes were normalized to GAPDH mRNA in the same sample. The data shown represent the mean ± SD (*n* = 3). ***, *P* < 0.0001. *P* value was analyzed by Sidak’s test

Given that TNFα induces MMP-9 expression in many types of cancer [31, 32], we wondered whether EA/EuH inhibits TNFα-induced expression of MMP-9 in various breast cancer cells. RT-PCR analysis showed that treatment with 10 ng/mL TNFα upregulated MMP-9 mRNA expression in MDA-MB-231, MDA-MB-435, T47D, and MCF7 human breast cancer cells, while this induction was substantially abolished in the presence of 5 μg/mL EA/EuH (Fig. 4a). To precisely quantify the changes in MMP-9 mRNA expression, quantitative real-time PCR analysis was performed (Fig. 4b). Treatment with 10 ng/mL TNFα alone resulted in 330 ± 65.6-, 313 ± 35.1-, 186 ± 61.2, and 233 ± 56.9-fold increases in MMP-9 mRNA levels in MDA-MB-231, MDA-MB-435, T47D, and MCF7 cells, respectively. However, these were significantly decreased to 75.7 ± 27.2-, 35.0 ± 8.89-, 16.3 ± 7.57-, and 22.7 ± 9.71-fold increases upon pre-treatment with 5 μg/mL EA/EuH, respectively (all *P* < 0.01 by Sidak’s test). These data suggest that EA/EuH can downregulate TNFα-induced MMP-9 mRNA expression in various breast cancer cells.

### EA/EuH inhibits TNFα-induced MMP-9 expression at the promoter level

**Fig. 5:**
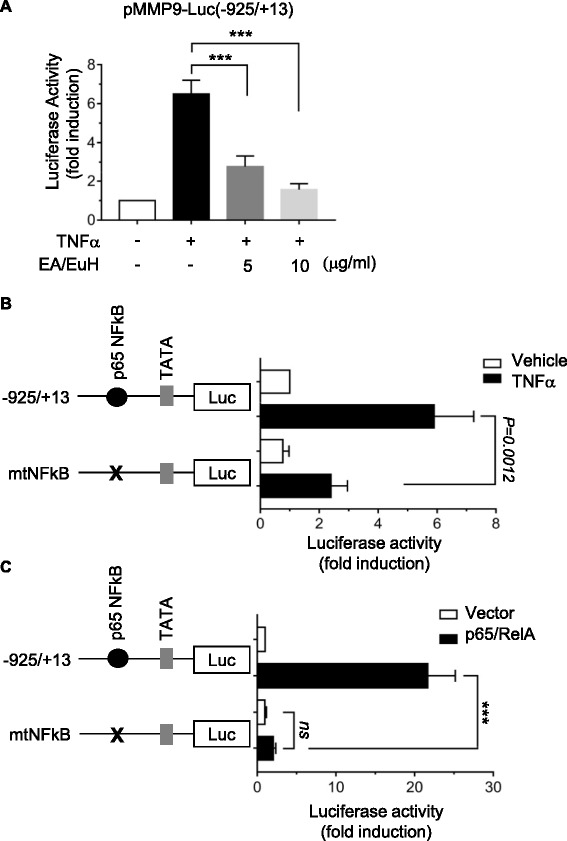
Inhibitory effect of EA/EuH on TNFα-induced MMP-9 promoter activation. **a** Promoter assay. MDA-MB-231 cells were transfected with 0.2 μg of promoter reporter, pMMP9-Luc(−925/+13). After 48 h, cells were treated with Ea/EuH (5 μg/mL) for 8 h, and their luciferase activities were measured. Firefly luciferase activity was normalized to Renilla luciferase activity. The data shown represent the mean ± SD (*n* = 9). ***, *P* < 0.0001. *P* value was analyzed by Sidak’s multiple comparisons test. **b** Either pMMP9-Luc(−925/+13) or pMMP9-Luc(−925/+13)mtNFκB was transfected into MDA-MB-231 cells. After 48 h, cells were treated with TNFα (10 ng/mL) for 8 h, and their luciferase activities were measured. The data shown represent the mean ± SD (*n* = 9). *P* value was analyzed by Sidak’s test. **c** Either pMMP9-Luc(−925/+13) or pMMP9-Luc(−925/+13)mtNFκB was co-transfected along with RelA/p65 nuclear factor kappa B (NF-κB) expression plasmid into MDA-MB-231 cells. After 48 h, cells were collected, and their luciferase activities were measured. The data shown represent the mean ± SD (n = 9). *ns*, not significant. ***, *P* < 0.0001. *P* value was analyzed by Sidak’s multiple comparisons test

To understand the molecular mechanism underlying EA/EuH-induced downregulation of MMP-9 mRNA expression, we examined the effect of EA/EuH on MMP-9 gene promoter activity. MDA-MB-231 cells were transfected with MMP-9 promoter reporter, pMMP9-Luc(−925/+13), and the luciferase reporter activity was assessed. We found that TNFα-induced MMP-9 promoter activity was significantly inhibited in a dose-dependent manner by 5 μg/mL EA/EuH treatment (all *P* < 0.0001 by Sidak’s test; Fig. 5a), indicating that EA/EuH reduced MMP-9 expression at the transcriptional level. The MMP-9 gene is regulated by multiple regulatory proteins, including NF-κB [32]. We also found that disruption of the NF-κB-binding site by site-directed mutagenesis abrogated TNFα-induced MMP-9 gene promoter activity (Fig. 5b) and RelA/p65 NF-κB-induced MMP-9 gene promoter activity (Fig. 5c). Thus, it is possible that EA/EuH-induced downregulation of MMP-9 expression is associated with the inhibition of NF-κB transcriptional activity.

### EA/EuH attenuates TNFα-induced NF-κB activation

**Fig. 6:**
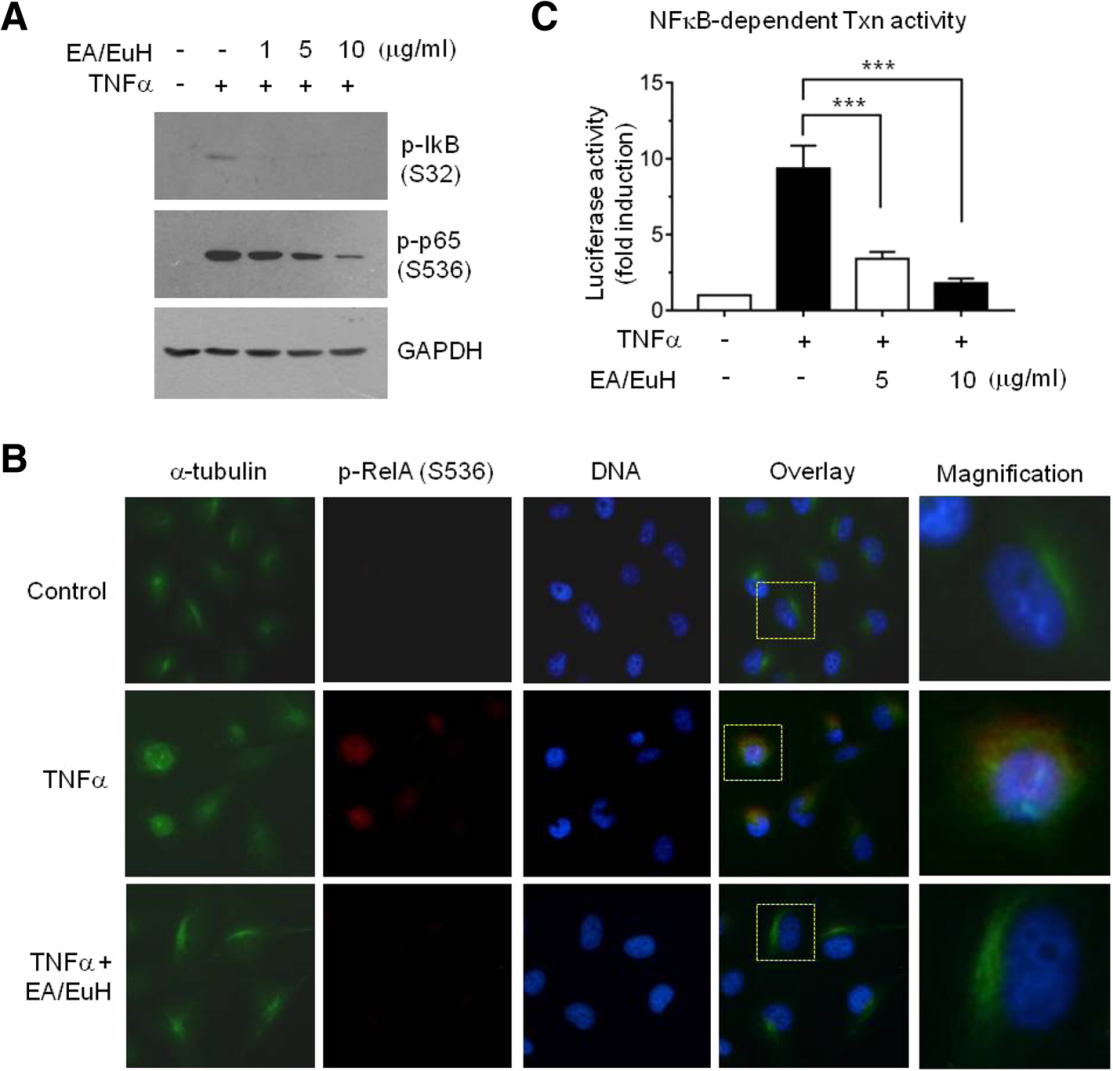
Inhibitory effect of EA/EuH on TNFα-induced NF-κB activation. **a** Serum-starved MDA-MB-231 cells were pre-treated with EA/EuH (5 μg/mL) for 30 min before stimulation with TNFα (10 ng/mL). After 20 min, whole-cell lysates were prepared, and immunoblotting was performed using the phospho-specific antibody against IκBα (Ser32) or RelA/p65 (Ser536) as indicated. The anti-GAPDH antibody was used as an internal control. **b** MDA-MB-231 cells were pre-treated with EA/EuH (5 μg/mL) for 30 min before stimulation with TNFα (10 ng/mL). After 20 min, the cells were fixed and incubated with antibodies against α-tubulin or phospho-RelA/p65 (Ser536) for 2 h, followed by incubation with Alexa Fluor 488-conjugated (*green signal*) or Alexa Fluor 555-conjugated (*red signal*) secondary antibody for 30 min. Nuclear DNA was stained with 1 μg/mL Hoechst 33258 for 10 min (*blue signal*). **c** MDA-MB-231 cells were transfected with 5× NFκB-Luc plasmid along with 50 ng pRL-null. At 48 h post-transfection, the cells were treated with or without 10 ng/mL TNFα in the absence or presence of EA/EuH (5 μg/mL). The data shown represent the mean ± SD. ***, *P* < 0.0001 (*n* = 9). *P* value was analyzed by Sidak’s multiple comparisons test

To investigate whether EA/EuH could inhibit NF-κB, MDA-MB-231 cells were treated with 10 ng/mL TNFα for 20 min in the absence or presence of EA/EuH. Phosphorylation status of IκB and RelA/p65 NF-κB was analyzed by immunoblot analysis. Treatment with EA/EuH dose-dependently inhibited TNFα-induced phosphorylation of IκB on serine-32 and RelA/p65 on serine-536 (Fig. 6a), suggesting that EA/EuH blocked the signaling pathway regulating IκB upstream kinase. To address further the inhibitory effect of EA/EuH on NF-κB, RelA/p65 phosphorylation was analyzed in MDA-MB-231 cells using immunofluorescent microscopy. Fluorescent staining for phospho-RelA at serine-536 was evident in the perinucleus and nucleus upon TNFα stimulation, which was suppressed by EA/EuH treatment (Fig. 6b). We next wondered whether EA/EuH-induced inhibition of NF-κB phosphorylation is functionally linked to the inhibition of transcriptional activity. NF-κB-dependent transcription was measured using a NF-κB *cis*-acting luciferase report system. Upon 10 ng/mL TNFα stimulation, NF-κB transcriptional activity was increased 9.3 ± 1.5-fold; however, this increase in transcriptional activity was significantly decreased in the presence of 5 μg/mL EA/EuH (all *P* < 0.0001 by Sidak’s test; Fig. 6c), suggesting that EA/EuH inhibits NF-κB-regulated gene transcription.

## Conclusion

*E. humifusa* Willd has been used in traditional medicine in the eastern Asia, including Korea and China, for a long time. This study demonstrates that the ethyl acetate fraction from *E. humifusa* Willd (EA/EuH) has a preventive effect on TNFα-induced invasive capability of highly metastatic MDA-MB-231 breast cancer cells through the inhibition of NF-κB-mediated MMP-9 gene expression. We also show that EA/EuH attenuated experimental lung metastasis in vivo as revealed by syngenic intra-splenic transplantation model of 4 T1 mouse mammary carcinoma cells. These findings suggest that *E. humifusa* Willd may be beneficial in the prevention of invasion and metastasis of early stage breast cancer.

## Abbreviations

EA: Ethyl acetate
ECM: Extracellular matrix
EuH: *Euphorbia humifusa* Willd
GAPDH: Glyceraldehyde-3-phosphate dehydrogenase
H&E: Hematoxylin and eosin
MMP: Matrix metalloproteinase
TNFα: Tumour necrosis factor alpha
